# RF Energy Harvesting and Information Transmission Based on NOMA for Wireless Powered IoT Relay Systems

**DOI:** 10.3390/s18103254

**Published:** 2018-09-27

**Authors:** Ashish Rauniyar, Paal Engelstad, Olav N. Østerbø

**Affiliations:** 1 Autonomous System and Networks Research Group, Department of Computer Science, Oslo Metropolitan University, 0130 Oslo, Norway; paalen@oslomet.no; 2 Autonomous Sensor and Technologies Research Group, Department of Technology Systems, University of Oslo, 0316 Oslo, Norway; 3 Telenor Research, 0316 Oslo, Norway; olav.osterbo@getmail.no

**Keywords:** Internet of Things, time switching, power splitting, NOMA, energy harvesting, radio frequency, relaying, outage probability, sum-throughput

## Abstract

Amidst the rapid development of the fifth generation (5G) networks, Internet of Things (IoT) is considered as one of the most important part of 5G next generation networks as it can support massive object communications. These massive object communications in the context of IoT is expected to consume a huge power. Furthermore, IoT sensors or devices are rather power constrained and are mostly battery operated. Therefore, energy efficiency of such network of IoT devices is a major concern. On the other hand, energy harvesting (EH) is an emerging paradigm that allows the wireless nodes to recharge themselves through radio frequency (RF) signals directed to them from the source node and then relaying or transmitting the information. Although a myriad of works have been carried out in the literature for EH, the vast majority of those works only consider RF EH at the relay node and successfully transmitting the source node data. Those approaches do not consider the data transmission of the relay node that may be an energy deprived IoT node which needs to transmit its own data along with the source node data to their respective destination nodes. Therefore, in this paper, we envisioned a RF EH and information transmission system based on time switching (TS) relaying, power splitting (PS) relaying and non-orthogonal multiple access (NOMA) which is suitable for wireless powered IoT relay systems. A source node information data is relayed through power constrained IoT relay node $IoT_{R}$ that first harvests the energy from source node RF signal using either TS and PS relaying protocol and then transmits the source node information along with its information using NOMA protocol to the respective destination nodes. Considering NOMA as a transmission protocol, we have mathematically derived analytical expressions for TS and PS relaying protocol for our proposed system. We have also formulated an algorithm to find out optimal TS and PS factor that maximizes the sum-throughput for our proposed system. Our proposed system analytical results for TS and PS protocol are validated by the simulation results.

## 1. Introduction

The Internet of Things (IoT) is a promising technology that aims to provide connectivity solutions. With the expeditious expansion of IoT technology across the globe, it is expected that billions of small sensors or devices will be connected with each other over the next few years [1,2,3]. The technological development in IoT integrates various sensors, devices, smart objects to be fully operated as autonomous device-to-device (D2D), machine-to machine (M2M) without any human intervention [4,5,6]. IoT is considered as one of the most important part of the fifth generation (5G) wireless systems as it can support massive object communications [7,8]. These massive object communications in the context of IoT is expected to consume a huge power. Therefore, energy efficient green communication within the context of 5G and IoT is a challenging problem to be solved [9].

Sensor nodes are the principal components which brings the idea of IoT into reality [10]. These massive IoT sensor nodes and devices are usually battery operated and hence replacement of battery in such small objects is not a feasible option. Moreover, cooperative communication has been widely studied to mitigate wireless impairments such as fading and other environmental factors [11,12,13,14]. However, conventional cooperative relaying techniques requires the participating relaying nodes to spend extra energy for data transmission which may prevent the battery operated IoT nodes to take an active part in relaying. Therefore, wireless energy harvesting (EH) from ambient Radio Frequency (RF) signals is considered as a buoyant energy efficient solution to combat the issue of powering massive IoT sensor and devices [15,16,17].

RF EH is thus considered as an appealing solution in extending the lifetime of these IoT sensors and devices from months to years and even decades, that ultimately enable their self-sustaining operations [18]. In wireless communication systems, simultaneous information and power transfer (SWIPT) is another emerging paradigm that allows the wireless nodes to recharge themselves through RF signals directed to them from the source node and then relaying or transmitting the information [19]. Meanwhile, accommodating multiple users that can be multiplexed in power domain, non-orthogonal multiple access (NOMA) has been proposed as another important candidate for future 5G technology for providing spectral efficiency and power gains [20,21]. The main idea of NOMA is to serve multiple users in the same frequency band, but with different power levels, which is fundamentally different from conventional orthogonal multiple access schemes [22]. In particular, power-domain NOMA allocates more transmit power to users with worse channel conditions and less transmitting power to users with better channel conditions in order to achieve a balanced trade-off between system throughput and user fairness. Therefore, users can be separated by successive interference cancellation (SIC) at the receiver side [23].

**Figure 1:**
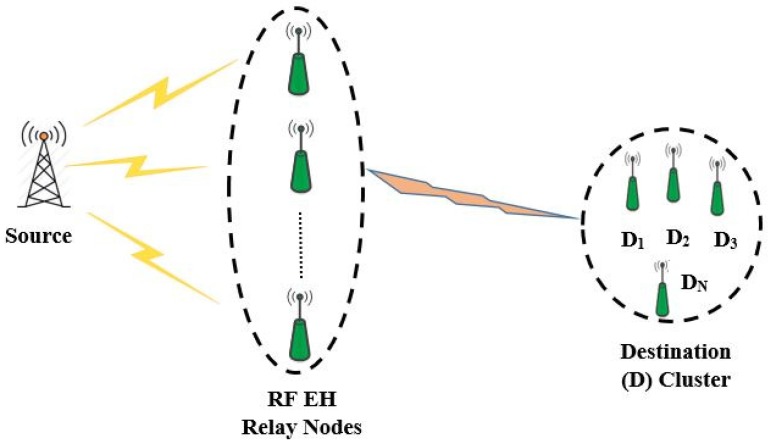
Generic RF EH relay communication system.

An illustration of generic RF EH relay communication system is shown in Figure 1, where a source node selects one of the RF EH relaying node to transmits its information to its intended destination. The harvested energy from RF source signals allows the relay node to power up themselves for simultaneous information processing and transmission (SWIPT) [24]. It is also understood that using more than one relay increases the complexity of the systems greatly [25]. Such cooperative RF EH relay communication systems as depicted by Figure 1, only considers the transmission of source node data successfully. In this paper, we envisioned an ubiquitous IoT relay system where an IoT node that can acts as a relay for transmitting source node information data to its intended destination and at the same time, it also transmits its own data to its destination node based on NOMA protocol. Furthermore, if EH is employed in such IoT relay systems, it has the potential to provide unlimited energy to sensor nodes and thus enabling self-sustainable green communications [26]. Also, in order for small IoT device to communicate and transmit data, M2M relaying has been proposed as a suitable heterogeneous architecture for 802.16p IoT, Third Generation Partnership Project (3GPP) machine type communications (MTC) and European Telecommunications Standards Institute (ETSI) M2M communication [27]. Hence, we believe that our considered scenario for IoT relay EH system fits to the standardization activities of ETSI and 3GPP projects for self-sustainable green communications.

In SWIPT, time splitting (TS) relaying and power splitting (PS) relaying schemes are very popular for energy harvesting and decoding the information separately. In TS relaying scheme, the receiver switches between energy harvesting and information decoding over time. However, in PS relaying scheme, the receiver uses a portion of received power for energy harvesting purpose and then uses the remaining power for information decoding.

Nasir et al. studied amplify-and-forward (AF) relaying network based on TS and PS relaying schemes [28]. They derived the analytical expressions for outage probability and the ergodic capacity for delay-limited and delay tolerant transmission modes. Du et al. investigated outage analysis of multi-user cooperative transmission network with TS and PS relay receiver architectures [29]. They theoretically analyze the system outage probability based on TS and PS relaying protocols. A cooperative SWIPT NOMA protocol has been studied in [30]. Here, near NOMA users that are close to source node acts as EH-based relay to help far NOMA users. Considering user selection schemes, they derived the closed-form expressions for the outage probability and system throughput. Ha et al. [31] studied the outage performance of EH-based decode-and-forward (DF) relaying NOMA networks by deriving the closed form equation of the outage probability. Two copies of same information from the source node direct link and EH-based relay link were received at the destination nodes. Kader et al. [32] studied TS and PS with EH and NOMA in a spectrum sharing environment. The secondary transmitter acts as an EH-based relay and then transmits the primary transmitter data along with its data using NOMA protocol. Jain et al. [33] also proposed an EH-based spectrum sharing protocol for wireless sensor networks. However, although a myriad of such EH works have been carried out in the literature, EH considering the energy-efficient data transmission of source and IoT relay node together based on TS, PS and NOMA suitable for IoT relay systems has not been considered in the previous works. This motivated us to propose an RF EH and information transmission based on TS, PS and NOMA for IoT relay systems and analyze their performance by deriving the analytical expressions for outage probability, throughput and sum-throughput.

In summary, the main contribution of this paper is as follows:

- Realizing the energy constrained nature of IoT nodes, we have considered and investigated an RF EH-based on TS, PS and NOMA for IoT relay systems.
- Although a myriad of works have been carried out in the literature for EH, the absolute vast majority of those works only consider RF EH at relay node and transmission of source node data successfully to its destination node. Those approaches do not consider the data transmission of the relay node that may be an IoT node which needs to transmit its data along with the source node data to their respective destinations. In this paper, we rather focus on RF EH and information transmission based on TS, PS relaying and NOMA for IoT relay systems.
- We have mathematically derived the outage probability, throughput and sum-throughput for our proposed system. We have also formulated an iterative algorithm-Golden Section Search Method to find the optimal time switching and power splitting factor for sum-throughput maximization.
- Our proposed system analytical results for TS and PS are validated by simulation results. The developed analysis is corroborated through Monte-Carlo simulations and some representative performance comparisons are presented.

The rest of the paper is organized as follows. In Section 2, we present the system model for the considered scenario. Section 3 deals with the considered system model based on time switching and NOMA protocol along with outage probability, throughput and sum-throughput derivations. Section 4 deals with the considered system model based on power splitting and NOMA protocol along with outage probability, throughput and sum-throughput derivations. In Section 5, we explain the algorithm—Golden Section Search Method to find out the optimal time switching and power splitting factor that maximizes the sum-throughput for our proposed system. Numerical results and discussions are presented in Section 6. Conclusions and future works are drawn in Section 7.

## 2. System Model

**Figure 2:**
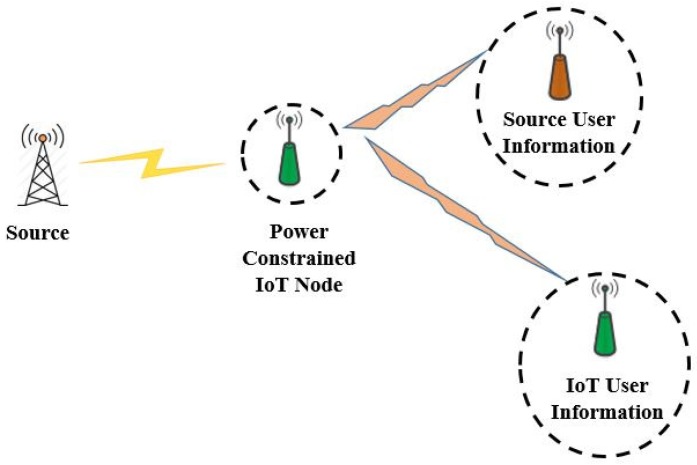
Considered system model scenario.

We have considered a cooperative relaying EH scenario as shown in Figure 2, where a source has to transmit its information data to the destination. Due to fading or weak link between a source-destination pair, the source node seek the help of IoT relay node ($IoT_{R}$) for relaying its information data. Here, the source node may be an IoT node which has abundant energy supply from the other sources. Cooperative communication with single relay is a simple but effective communication scheme especially for energy constrained networks such as IoT networks [34]. Furthermore, using more than one relay increases the complexity of the systems greatly [25]. Therefore, we have considered a single $IoT_{R}$ node for our system model. However, it can be extended to multiple $IoT_{R}$ node scenario as well.

$IoT_{R}$ is rather power constrained node that acts as a DF relay. It first harvests the RF energy from source signal using either time switching protocol or power splitting protocol in the first stage and then transmits the source information data along with its own data using NOMA protocol in next subsequent stage. The dual purpose of energy harvesting and forwarding the information data is thus served by $IoT_{R}$. The receiving end for source and $IoT_{R}$ node serves as the destination for data transmission. Unlike several of the previous works, here the information data forwarded by $IoT_{R}$ node is the source node information data and its own data.

## 3. System Model Based on Time Switching and NOMA

**Figure 3:**
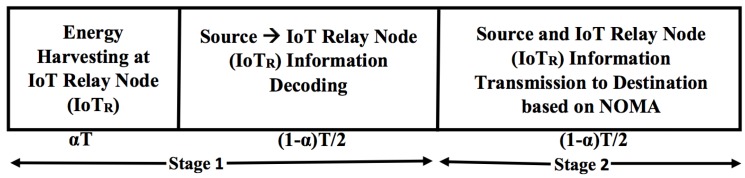
System model based on time switching and NOMA.

The proposed system model based on TS and NOMA is shown in Figure 3. In this TS relaying scheme, power constrained $IoT_{R}$ node first harvests the energy from the source node’s RF signal for αT duration and uses the time $\frac{(1-\alpha )T}{2}$ for information processing and $\frac{(1-\alpha )T}{2}$ for information transmission to the source and IoT user using NOMA protocol. We have assumed that all nodes are considered to be operating in half duplex mode. An independent Rayleigh block fading with channel coefficient $h_{i}∼CN\left(0,\lambda_{i}=d_{i}^{-v}\right)$ with zero mean and variance $\lambda_{i}$ is assumed between any two nodes where, $d_{i}$ is the distance between the corresponding link and *v* is the path loss exponent.The detailed step of our proposed system model based on TS and NOMA is given below.

### 3.1. Stage 1

In this stage, the source transmits signal $x_{s}$ with power $P_{s}$ to the $IoT_{R}$ for half of the block time *T* i.e., T/2 period of time. Here, $IoT_{R}$ node works as TS-based relay. The $IoT_{R}$ node divide the time block in the ratio $\alpha T:\frac{(1-\alpha )T}{2}:\frac{(1-\alpha )T}{2}$. Here αT is for energy harvesting by $IoT_{R}$ and $\frac{(1-\alpha )T}{2}$ is for information processing by $IoT_{R}$ respectively, 0≤α≤1. The information signal received at $IoT_{R}$ during this stage is given as:

(1) $${\hat{y}}_{IoT_{R}}=\sqrt{P_{s}}h_{IoT_{R}}x_{s}+n_{IoT_{R}},$$

where $n_{IoT_{R}}∼CN\left(0,\sigma_{IoT_{R}}^{2}\right)$ is the additive white Gaussian noise at $IoT_{R}$ with mean zero and variance $\sigma_{IoT_{R}}^{2}$. $h_{IoT_{R}}∼CN\left(0,\lambda_{h}\right)$ is the channel coefficient between source node and $IoT_{R}$ node with zero mean and variance $\lambda_{h}$.

The energy harvested at $IoT_{R}$ in αT duration of time is given as:

(2) $${\hat{E}}_{h_{IoT_{R}}}=\eta P_{s}{\left|h_{IoT_{R}}\right|}^{2}\alpha T,$$

where 0≤η≤1 is the energy conversion efficiency. Here, we assume that the pre-processing power for the energy harvesting is negligible in contrast to the transmission power $P_{s}$ which is in line with the previous works [31,32,33].

The transmit power of $IoT_{R}$ i.e., ${\hat{P}}_{IoT_{R}}$ in $\frac{(1-\alpha )T}{2}$ block of time can be given as:

(3) $${\hat{P}}_{IoT_{R}}=\frac{{\hat{E}}_{h_{IoT_{R}}}}{(1-\alpha )T/2}=\frac{2\eta P_{s}{\left|h_{IoT_{R}}\right|}^{2}\alpha }{\left(1-\alpha \right)},$$

### 3.2. Stage 2

In this stage, the $IoT_{R}$ node transmits a superimposed composite signal ${\hat{Z}}_{I_{C1}}$ which consists of source information $x_{s}$ and $IoT_{R}$ information $x_{IoT_{R}}$ to the respective destination of source and IoT relay node using NOMA protocol. The superimposed composite signal ${\hat{Z}}_{I_{C1}}$ following NOMA protocol can be given as:

(4) $${\hat{Z}}_{I_{C1}}=\sqrt{\phi_{1}{\hat{P}}_{IoT_{R}}}x_{s}+\sqrt{\phi_{2}{\hat{P}}_{IoT_{R}}}x_{IoT_{R}}$$

where $\phi_{1}+\phi_{2}=1$ and $\phi_{2}=1-\phi_{1}$ is the power allocation factor for the NOMA protocol.

Now, the received signals at the receiver of Source user and IoT user can be respectively given as:

(5) $${\hat{y}}_{s_{rec}}=\sqrt{{\hat{P}}_{IoT_{R}}}h_{s_{rec}}{\hat{Z}}_{I_{C1}}+n_{s_{rec}},$$

(6) $${\hat{y}}_{IoT_{rec}}=\sqrt{{\hat{P}}_{IoT_{R}}}h_{IoT_{rec}}{\hat{Z}}_{I_{C1}}+n_{IoT_{rec}},$$

where $n_{s_{rec}}$ and $n_{IoT_{rec}}$ is the additive white Gaussian noise at the receiver of source and IoT user node respectively with mean zero and variance $\sigma_{s_{rec}}^{2}$ and $\sigma_{IoT_{rec}}^{2}$. Also, $h_{s_{rec}}∼CN\left(0,\lambda_{g}\right)$ is the channel coefficient between $IoT_{R}$ node and receiving source user with zero mean and variance $\lambda_{g}$ and $h_{IoT_{rec}}∼CN\left(0,\lambda_{z}\right)$ is the channel coefficient between $IoT_{R}$ node and receiving IoT user with zero mean and variance $\lambda_{z}$. We have also assumed that $h_{s_{rec}}>h_{IoT_{rec}}$. Therefore, $\lambda_{g}>\lambda_{z}$ and $\phi_{1}<\phi_{2}$.

### 3.3. Outage Probability, Throughput and Sum-Throughput

According to Equation (1), the received signal to noise ratio (SNR) at $IoT_{R}$ is given by:

(7) $${\hat{\gamma }}_{IoT_{R}}=\frac{P_{s}{\left|h_{IoT_{R}}\right|}^{2}}{\sigma_{IoT_{R}}^{2}}=\hat{\delta }{\left|h_{IoT_{R}}\right|}^{2}$$

where $\hat{\delta }≜\frac{P_{s}}{\sigma_{IoT_{R}}^{2}}$ represents the transmit signal-to-noise ratio (SNR) from the source.

According to Equation (4), the received SNR with $x_{IoT_{R}}$ and $x_{s}$ at the receiving source user is given by:

(8) $${\hat{\gamma }}_{s_{rec}}^{x_{IoT_{R}}\to x_{s}}=\frac{\phi_{2}{\hat{P}}_{IoT_{R}}{\left|h_{s_{rec}}\right|}^{2}}{\phi_{1}{\hat{P}}_{IoT_{R}}{\left|h_{s_{rec}}\right|}^{2}+\sigma_{s_{rec}}^{2}}$$

(9) $${\hat{\gamma }}_{s_{rec}}=\frac{\phi_{1}{\hat{P}}_{IoT_{R}}{\left|h_{s_{rec}}\right|}^{2}}{\sigma_{s_{rec}}^{2}}$$

where ${\hat{\gamma }}_{s_{rec}}^{x_{IoT_{R}}\to x_{s}}$ is the SNR required at $x_{s}$ to decode and cancel $x_{IoT_{R}}$.

The received SNR at IoT user associated with symbol $x_{IoT_{R}}$ is given by:

(10) $${\hat{\gamma }}_{IoT_{rec}}=\frac{\phi_{2}{\hat{P}}_{IoT_{R}}{\left|h_{IoT_{rec}}\right|}^{2}}{\phi_{1}{\hat{P}}_{IoT_{R}}{\left|h_{IoT_{rec}}\right|}^{2}+\sigma_{IoT_{rec}}^{2}}$$

As we can see from Figure 2, the data transmission is break down into two separate hops which are independent of each other. Hence, the outage occurs only if source to $IoT_{R}$ path and $IoT_{R}$ to corresponding destination path fails to satisfy the SNR constraint. Therefore, the outage probability of the source can be given as:

(11) $${\hat{P}}_{Out_{S}}=Pr\left(min\left({\hat{\gamma }}_{IoT_{R}},{\hat{\gamma }}_{s_{rec}}\right)\leq \hat{\psi }\right)$$

where $\hat{\psi }=2^{R}-1$ is the lower threshold for SNR i.e., outage probability.

Similarly, the outage probability of the IoT relay node $IoT_{R}$ can be given as:

(12) $${\hat{P}}_{Out_{IoT_{R}}}=Pr\left(min\left({\hat{\gamma }}_{s_{rec}}^{x_{IoT_{R}}\to x_{s}},{\hat{\gamma }}_{IoT_{rec}}\right)\leq \hat{\psi }\right)$$

The throughput of the source node can be given as:

(13) $${\hat{Thr}}_{S}=\frac{\left(1-{\hat{P}}_{out_{S}}\right)\left(1-\alpha \right)R}{2}$$

where *R* is the transmission rate in bits per second per hertz.

The throughput of the IoT relay node $IoT_{R}$ can be given as:

(14) $${\hat{Thr}}_{IoT_{R}}=\frac{\left(1-{\hat{P}}_{Out_{IoT_{R}}}\right)\left(1-\alpha \right)R}{2}$$

Therefore, the sum-throughput of the whole system using TS and NOMA can be given as:

(15) $$\begin{array}{cc} \hat{Thr} & ={\hat{Thr}}_{S}+{\hat{Thr}}_{IoT_{R}}=\frac{\left(1-{\hat{P}}_{Out_{S}}\right)\left(1-\alpha \right)R}{2}+\frac{\left(1-{\hat{P}}_{Out_{IoT_{R}}}\right)\left(1-\alpha \right)R}{2} \end{array}$$

**Theorem** **1.** *The outage probability and throughput of the source node using TS and NOMA can be expressed as:*

(16) $$\begin{array}{c} {\hat{P}}_{{Out}_{S}}=1-2\sqrt{\frac{\lambda_{h}\lambda_{g}x_{0}}{k}}K_{1}\left(2\sqrt{\frac{\lambda_{h}\lambda_{g}x_{0}}{k}}\right)+\sum_{n=0}^{\infty }\frac{{\left(-1\right)}^{n}}{n\;!}{\left(\lambda_{h}x_{0}\right)}^{n+1}E_{n+2}\left(\frac{\lambda_{g}}{k}\right) \end{array}$$

(17) $$\begin{array}{c} {\hat{Thr}}_{S}=\frac{R(1-\alpha )}{2}\left(2\sqrt{\frac{\lambda_{h}\lambda_{g}x_{0}}{k}}K_{1}\left(2\sqrt{\frac{\lambda_{h}\lambda_{g}x_{0}}{k}}\right)-\sum_{n=0}^{\infty }\frac{{\left(-1\right)}^{n}}{n\;!}{\left(\lambda_{h}x_{0}\right)}^{n+1}E_{n+2}\left(\frac{\lambda_{g}}{k}\right)\right) \end{array}$$

*where, $x_{0}=\frac{\hat{\psi }}{\hat{\delta }}$, $k=\frac{2\alpha \eta \phi_{1}}{\left(1-\alpha \right)}$, $K_{1}\left(.\right)$ is a first-order modified Bessel function of the second kind, and $E_{n}\left(a\right)=\int_{y=1}^{\infty }y^{-n}e^{-ay}dy$ is the exponential integral of order n.*

**Proof.**  The detailed proof is given in Appendix A. ☐

**Theorem** **2.** *The outage probability and throughput of the IoT relay node using TS and NOMA can be expressed as:*

(18) $${\hat{P}}_{Out_{IoT_{R}}}=1-2\sqrt{d\lambda_{h}\left(\lambda_{g}+\lambda_{z}\right)}K_{1}\left(2\sqrt{d\lambda_{h}\left(\lambda_{g}+\lambda_{z}\right)}\right)$$

(19) $${\hat{Thr}}_{IoT_{R}}=\frac{R(1-\alpha )}{2}\left(2\sqrt{d\lambda_{h}\left(\lambda_{g}+\lambda_{z}\right)}K_{1}\left(2\sqrt{d\lambda_{h}\left(\lambda_{g}+\lambda_{z}\right)}\right)\right)$$

*where, $d=\frac{\hat{\psi }}{(\phi_{2}-\phi_{1}\hat{\psi })l}$, $l=\frac{2\alpha \eta P_{s}}{\left(1-\alpha \right)}$*

**Proof.**  The detailed proof is given in Appendix B. ☐

Combining Equations (17) and (19), we finally get the analytical equation for the sum-throughput of the proposed system using TS and NOMA.

## 4. System Model Based on Power Splitting and NOMA

**Figure 4:**
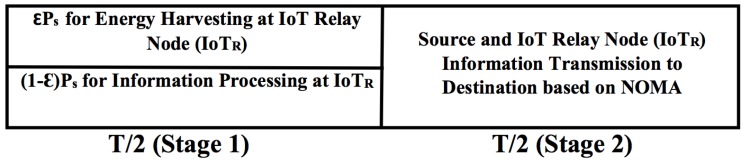
System model based on power splitting and NOMA.

The proposed system model based on PS and NOMA protocol is shown in Figure 4. In this PS relaying scheme, power constrained ($IoT_{R}$) node first harvests the energy from the source node signal using $\epsilon P_{s}$ where $P_{s}$ is the power of the source transmit signal. $IoT_{R}$ uses remaining power $\left(1-\epsilon \right)P_{s}$ for information processing.

### 4.1. Stage 1

During this stage, a source node signal $x_{s}$ with $P_{s}$ power is transmitted to the $IoT_{R}$ node for half of the block time *T* i.e., T/2 period of time. The $IoT_{R}$ node divide the received power $P_{s}$ in the ratio $\varepsilon P_{s}:(1-\varepsilon )P_{s}$. Accordingly here, $\varepsilon P_{s}$ is for energy harvesting and $\left(1-\varepsilon \right)P_{s}$ is for information processing by $IoT_{R}$ respectively, 0≤ε≤1. The information signal received at $IoT_{R}$ during this stage is given as:

(20) $$y_{IoT_{R}}=\sqrt{P_{s}}h_{IoT_{R}}x_{s}+n_{IoT_{R}},$$

The energy harvested at $IoT_{R}$ in T/2 period of time is given as:

(21) $$E_{h_{IoT_{R}}}=\frac{\eta \varepsilon P_{s}{\left|h_{IoT_{R}}\right|}^{2}T}{2},$$

The signal received at the information receiver of the $IoT_{R}$ is given as:

(22) $$\sqrt{\left(1-\varepsilon \right)}y_{IoT_{R}}=\sqrt{\left(1-\varepsilon \right)P_{s}}h_{IoT_{R}}x_{s}+n_{IoT_{R}},$$

The transmit power of $IoT_{R}$ i.e., $P_{IoT_{R}}$ in T/2 block of time is given as:

(23) $$P_{IoT_{R}}=\frac{E_{h_{IoT_{R}}}}{T/2}=\eta \varepsilon P_{s}{\left|h_{IoT_{R}}\right|}^{2},$$

### 4.2. Stage 2

In this stage, the $IoT_{R}$ node transmits a superimposed composite signal $Z_{I_{C1}}$ which consists of source information $x_{s}$ and $IoT_{R}$ information $x_{IoT_{R}}$ to the respective destination node i.e., source user and IoT user using NOMA protocol. The superimposed composite signal $Z_{I_{C1}}$ following NOMA protocol is given as:

(24) $$Z_{I_{C1}}=\sqrt{\phi_{1}P_{IoT_{R}}}x_{s}+\sqrt{\phi_{2}P_{IoT_{R}}}x_{IoT_{R}}$$

where $\phi_{1}+\phi_{2}=1$ and $\phi_{2}=1-\phi_{1}$.

Now, the received signals at the respective source user and IoT user can be given as:

(25) $$y_{s_{rec}}=\sqrt{P_{IoT_{R}}}h_{s_{rec}}Z_{I_{C1}}+n_{s_{rec}},$$

(26) $$y_{IoT_{rec}}=\sqrt{P_{IoT_{R}}}h_{IoT_{rec}}Z_{I_{C1}}+n_{IoT_{rec}},$$

### 4.3. Outage Probability, Throughput and Sum-Throughput

According to Equation (22), the received signal to noise ratio (SNR) at $IoT_{R}$ node is given by:

(27) $$\gamma_{IoT_{R}}=\frac{\left(1-\varepsilon \right)P_{s}{\left|h_{IoT_{R}}\right|}^{2}}{\sigma_{IoT_{R}}^{2}}=\left(1-\varepsilon \right)\delta {\left|h_{IoT_{R}}\right|}^{2}$$

where $\delta ≜\frac{P_{s}}{\sigma_{IoT_{R}}^{2}}$ represents the transmit signal-to-noise ratio (SNR) from the source.

According to Equation (25), the received SNR with $x_{IoT_{R}}$ and $x_{s}$ at the receiving source user is given by:

(28) $$\gamma_{s_{rec}}^{x_{IoT_{R}}\to x_{s}}=\frac{\phi_{2}P_{IoT_{R}}{\left|h_{s_{rec}}\right|}^{2}}{\phi_{1}P_{IoT_{R}}{\left|h_{s_{rec}}\right|}^{2}+\sigma_{s_{rec}}^{2}}$$

(29) $$\gamma_{s_{rec}}=\frac{\phi_{1}P_{IoT_{R}}{\left|h_{s_{rec}}\right|}^{2}}{\sigma_{s_{rec}}^{2}}$$

where $\gamma_{s_{rec}}^{x_{IoT_{R}}\to x_{s}}$ is the SNR required at the receiving source user to decode and cancel $IoT_{R}$ information i.e., $x_{IoT_{R}}$.

The received SNR at the receiving IoT user node associated with symbol $x_{IoT_{R}}$ is given by:

(30) $$\gamma_{IoT_{rec}}=\frac{\phi_{2}P_{IoT_{R}}{\left|h_{IoT_{rec}}\right|}^{2}}{\phi_{1}P_{IoT_{R}}{\left|h_{IoT_{rec}}\right|}^{2}+\sigma_{IoT_{rec}}^{2}}$$

As we can see from Figure 2, the data transmission is break down into two separate hops which are independent of each other. Hence, the outage occurs only if source to $IoT_{R}$ path and $IoT_{R}$ to corresponding destination path fails to satisfy the SNR constraint. Therefore, the outage probability of the source node can be given as:

(31) $$P_{Out_{S}}=Pr\left(min\left(\gamma_{IoT_{R}},\gamma_{s_{rec}}\right)\leq \psi \right)$$

where $\psi =2^{R}-1$ is the lower threshold for SNR i.e., outage probability, *R* being the target data rate.

Similarly, the outage probability of the $IoT_{R}$ node can be given as:

(32) $$P_{Out_{IoT_{R}}}=Pr\left(min\left(\gamma_{s_{rec}}^{x_{IoT_{R}}\to x_{s}},\gamma_{IoT_{rec}}\right)\leq \psi \right)$$

The throughput of the source node can be given as:

(33) $$Thr_{S}=\frac{(1-P_{Out_{S}})R}{2}$$

where *R* is measured in bits per second per hertz.

The throughput of the IoT relay node can be given as:

(34) $$Thr_{IoT_{R}}=\frac{(1-P_{Out_{IoT_{R}}})R}{2}$$

The factor 1/2 in Equations (33) and (34) is originated by the predicament that the two transmission phases are involved in the system.

Therefore, the sum-throughput of the whole system can be given as:

(35) $$Thr=Thr_{S}+Thr_{IoT_{R}}=\left(1-P_{Out_{S}}\right)\frac{R}{2}+\left(1-P_{Out_{IoT_{R}}}\right)\frac{R}{2}$$

**Theorem** **3.** *The outage probability and throughput of the source node using PS and NOMA can be expressed as:*

(36) $$\begin{array}{c} P_{{Out}_{S}}=1-2\sqrt{\frac{\lambda_{h}\lambda_{g}\left(1-\varepsilon \right)x_{0}}{a}}K_{1}\left(2\sqrt{\frac{\lambda_{h}\lambda_{g}\left(1-\varepsilon \right)x_{0}}{a}}\right)+\sum_{n=0}^{\infty }\frac{{\left(-1\right)}^{n}}{n\;!}{\left(\lambda_{h}x_{0}\right)}^{n+1}E_{n+2}\left(\frac{\left(1-\varepsilon \right)\lambda_{g}}{a}\right) \end{array}$$

(37) $$\begin{array}{c} Thr_{S}=\frac{R}{2}\left(2\sqrt{\frac{\lambda_{h}\lambda_{g}\left(1-\varepsilon \right)x_{0}}{a}}K_{1}\left(2\sqrt{\frac{\lambda_{h}\lambda_{g}\left(1-\varepsilon \right)x_{0}}{a}}\right)-\sum_{n=0}^{\infty }\frac{{\left(-1\right)}^{n}}{n\;!}{\left(\lambda_{h}x_{0}\right)}^{n+1}E_{n+2}\left(\frac{\left(1-\varepsilon \right)\lambda_{g}}{a}\right)\right) \end{array}$$

*where $x_{0}=\frac{\psi }{(1-\varepsilon )\delta }$, $a=\varepsilon \eta \phi_{1}$, $K_{1}\left(.\right)$ is a first-order modified Bessel function of the second kind, and $E_{n}\left(a\right)=\int_{y=1}^{\infty }y^{-n}e^{-ay}dy$ is the exponential integral of order n.*

**Proof.**  The detailed proof is formulated in Appendix C. ☐

**Theorem** **4.** *The outage probability and throughput of the IoT node using PS and NOMA can be expressed as:*

(38) $$P_{Out_{IoT_{R}}}=1-2\sqrt{c\lambda_{h}\left(\lambda_{g}+\lambda_{z}\right)}K_{1}\left(2\sqrt{c\lambda_{h}\left(\lambda_{g}+\lambda_{z}\right)}\right)$$

(39) $$Thr_{IoT_{R}}=\frac{R}{2}\left(2\sqrt{c\lambda_{h}\left(\lambda_{g}+\lambda_{z}\right)}K_{1}\left(2\sqrt{c\lambda_{h}\left(\lambda_{g}+\lambda_{z}\right)}\right)\right)$$

*where $c=\frac{\psi }{(\phi_{2}-\phi_{1}\psi )b}$, b=ηδε.*

**Proof.**  The detailed proof is formulated in Appendix D. ☐

Combining Equations (37) and (39), we finally get the analytical equation for the sum-throughput of the proposed system using PS and NOMA.

## 5. Optimal Time Switching $\alpha^{*}$ and Optimal Power Splitting Factor $\varepsilon^{*}$ for Sum-Throughput Maximization

To find out optimal time switching factor $\alpha^{*}$ and power splitting factor $\varepsilon^{*}$ that gives the best performance for sum-throughput maximization for our proposed system using TS, PS and NOMA, we evaluate ${\left(\frac{d\hat{Thr}\left(\alpha \right)}{d\alpha }\right)}_{TS}=0$ and ${\left(\frac{dThr(\varepsilon )}{d\varepsilon }\right)}_{PS}=0$, where $\hat{Thr}\left(\alpha \right)$ is the sum-throughput function with respect to time switching factor *α* and Thr(ε) is the sum-throughput function with respect to power splitting factor *ε* respectively. By analyzing the sum-throughput function for source and IoT node versus *α* and *ε*, we determine that this is concave function which has a unique maxima $\alpha^{*}$, $\varepsilon^{*}$ on the interval [0,1]. Therefore, we resort to Golden section search method [35] which is simple yet compelling iterative process to find out the optimal $\alpha^{*}$ and $\varepsilon^{*}$ that maximizes the sum-throughput of the proposed system using TS and PS respectively. The Golden section search method for determining optimal $\alpha^{*}$ and $\varepsilon^{*}$ is shown in Algorithm 1.


**Algorithm 1**
Golden Section Search Method for Finding Optimal Time Switching Factor $\alpha^{*}$ and Optimal Power Splitting Factor $\varepsilon^{*}$

**Input:***η*, *δ*, *R*, $\phi_{1}$, $\phi_{2}$**Initialization:** Set the start interval a=0.001, end interval b=0.99, golden proportion coefficient τ=0.618, the iteration index, accuracy value μ=0.000001, choose starting points $x_{1}=a+\left(1-\tau \right)*\left(b-a\right)$ and $x_{2}=a+\tau *\left(b-a\right)$**Output:** Optimal $\alpha^{*}$ and $\varepsilon^{*}$

 1:do function evaluation for respective TS and PS protocol i.e., ${\left(\frac{d\hat{Thr}\left(\alpha \right)}{d\alpha }\right)}_{TS}$, ${\left(\frac{dThr(\varepsilon )}{d\varepsilon }\right)}_{PS}$ at point $x_{1}$ and $x_{2}$ 2:
**repeat**
 3:*if* evaluated function ${\left(\frac{d\hat{Thr}\left(\alpha \right)}{d\alpha }\right)}_{x_{1}}<{\left(\frac{d\hat{Thr}\left(\alpha \right)}{d\alpha }\right)}_{x_{2}}$, ${\left(\frac{dThr(\varepsilon )}{d\varepsilon }\right)}_{x_{1}}<{\left(\frac{dThr(\varepsilon )}{d\varepsilon }\right)}_{x_{2}}$ then
 4:choose $b=x_{2}$, $x_{2}=x_{1}$ and find new point $x_{1}$ for both TS and PS 5:do function evaluation as step 1 6:
*else*
 7:
*$a=x_{1}$, $x_{1}=x_{2}$ and find new point $x_{2}$ for both TS and PS*
 8:do function evaluation as step 1 9:
*end if*
10:**until**|b−a|>μ and iteration index = maxChoosing Optimal $\alpha^{*}$ for TS11:*if* evaluated function ${\left(\frac{d\hat{Thr}\left(\alpha \right)}{d\alpha }\right)}_{x_{1}}<{\left(\frac{d\hat{Thr}\left(\alpha \right)}{d\alpha }\right)}_{x_{2}}$ then12:
$\alpha^{*}=x_{1}$
13:
*else*
14:
$\alpha^{*}=x_{2}$
Choosing Optimal $\varepsilon^{*}$ for PS15:*if* evaluated function ${\left(\frac{dThr(\varepsilon )}{d\varepsilon }\right)}_{x_{1}}<{\left(\frac{dThr(\varepsilon )}{d\varepsilon }\right)}_{x_{2}}$ then16:
$\varepsilon^{*}=x_{1}$
17:
*else*
18:
$\varepsilon^{*}=x_{2}$
19:end of Algorithm 1


## 6. Numerical Results and Discussion

**Figure 5:**
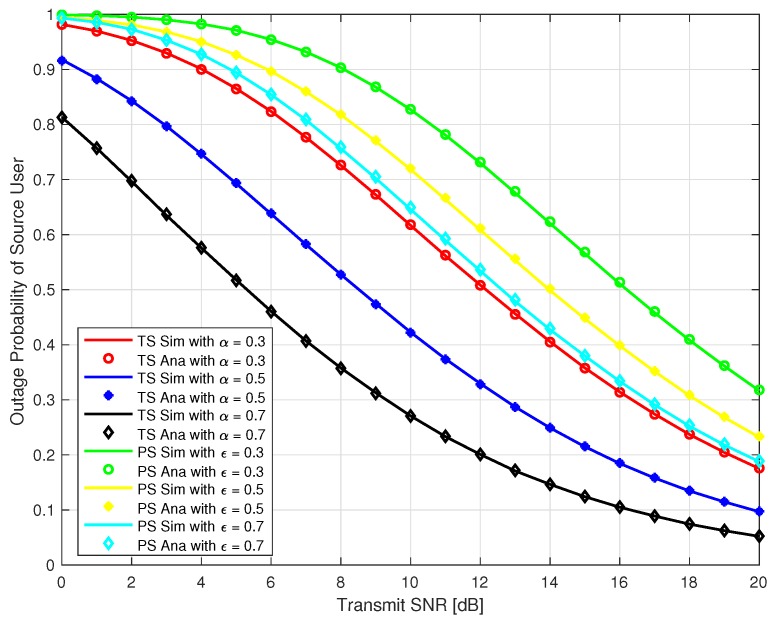
Outage Probability of Source User.

**Figure 6:**
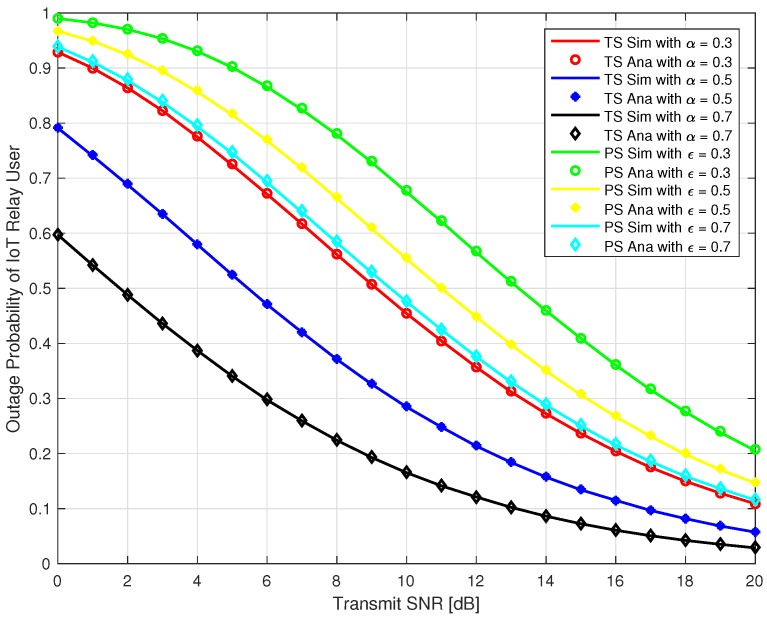
Outage Probability of IoT Relay User.

In this section, we present Monte-Carlo simulation results to verify our analysis for the proposed system as explained in the previous section for both TS and PS protocol. The simulation parameters are given in Table 1. We use MATLAB to run the Monte-Carlo simulation by averaging over $10^{5}$ random realizations of Rayleigh block fading channels $h_{IoT_{R}}$, $h_{s_{rec}}$, $h_{IoT_{rec}}$ and get the simulation results. In Figure 5 and Figure 6, the outage probability of the source user and IoT relay user are plotted against the transmit SNR at different time switching factor *α* = 0.3, 0.5, & 0.7 for TS relaying and different power splitting factor *ε* = 0.3, 0.5, & 0.7 for PS relaying. It can be observed that outage probability is a decreasing function with respect to increase in transmit SNR and *α* for TS protocol. It can also be observed that outage probability is also a decreasing function with respect to increase in transmit SNR and *ε* for PS protocol. Furthermore, our analysis exactly matched with the simulation results as depicted in Figure 5 and Figure 6. From Figure 5 and Figure 6, it should be noted that the outage probability of the source and IoT relay user using PS is higher than the TS protocol for our proposed system.

**Figure 7:**
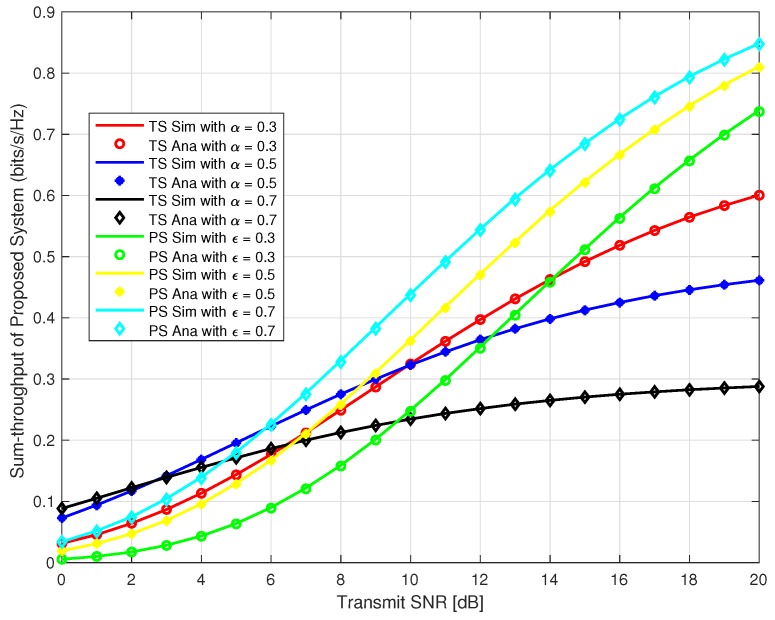
Sum-throughput of proposed system.

Considering, source user and IoT relay user as two user in the system for our proposed system, in Figure 7, we plotted the sum-throughput against the transmit SNR at time switching *α* = 0.3, 0.5, & 0.7 for TS and different power splitting factor *ε* = 0.3, 0.5, & 0.7 for PS. It can be observed that sum-throughput is a increasing function with respect to increase in transmit SNR and *α* for TS. Also, it is observed that sum-throughput is a increasing function with respect to increase in *δ* and *ε* for PS. Moreover, sum-throughput is higher for PS as compared to TS with the same varying amount of *α* and *ε* respectively for transmit SNR greater than 10 dB. At transmit SNR less than 6 dB, TS outperforms the PS protocol.

**Figure 8:**
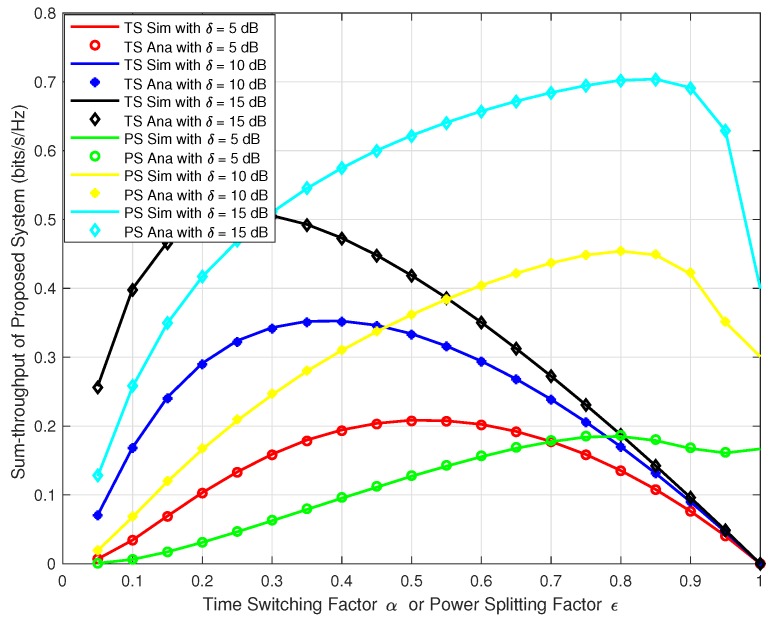
Sum-throughput of proposed system v/s *α* or *ϵ* with different *δ*.

**Figure 9:**
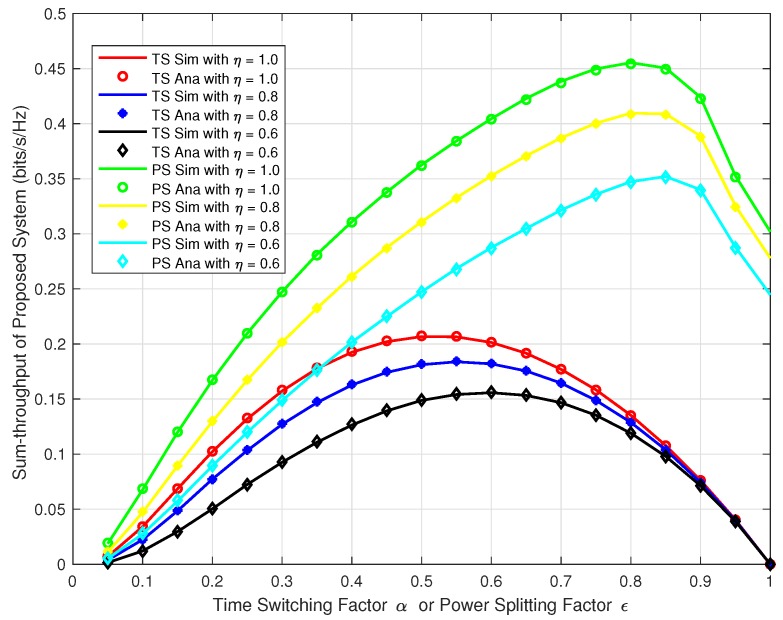
Sum-throughput of proposed system v/s *α* or *ϵ* with different *η*.

Next, we wanted to verify our analysis for the proposed system at different time switching factor *α* and power splitting factor *ε* for both TS and PS protocol. We plotted the sum-throughput against the *α* and *ε* varying from 0 to 1 and at *δ* = 5, 10, & 15. In Figure 8, we can observe the trend that, the sum-throughput first increases with the increase in *α*, *ε*, and *δ*, reaches to the maximum and then decreases. Similarly, in Figure 9, we plotted the sum-throughput for our proposed system with δ=10 at varying energy harvesting efficiency factor *η* = 0.6, 0.8, & 1.0 for both TS and PS. We can observe a similar trend as in Figure 8. The sum-throughput of the system first increases with the increase in *α*, *ε*, and *η*, reaches to the maximum and then decreases. This confirms that the sum-throughput is maximum at some optimal time switching factor $\alpha^{*}$ and optimal power splitting factor $\varepsilon^{*}$. In reality, we cannot have high *α* and *ε* as there will be less time and power allocated for information processing. Hence, there will be an outage in the system as no communication data will be transferred to the respective destinations.

**Figure 10:**
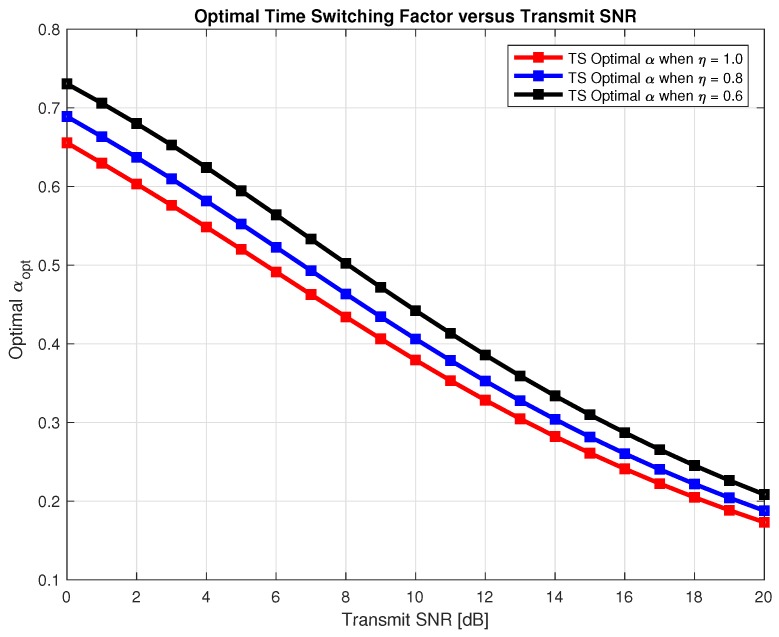
Optimal *α* for sum-throughput maximization.

**Figure 11:**
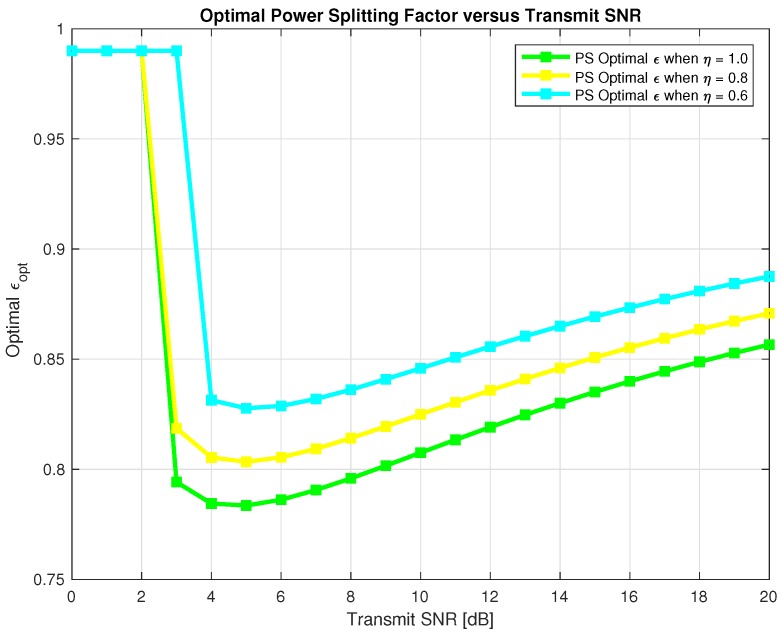
Optimal *ϵ* for sum-throughput maximization.

Therefore, we need to find optimal $\alpha^{*}$ and $\varepsilon^{*}$ that maximizes the sum-throughput for the proposed system for TS and PS respectively. In Figure 10 and Figure 11, we found out optimal $\alpha^{*}$ for TS and optimal $\varepsilon^{*}$ for PS respectively that maximizes the sum-throughput of the proposed system through Golden section search method as explained in Algorithm 1 and plotted it against the transmit SNR. In Figure 10, we can observe that optimal $\alpha^{*}$ linearly decreases with increase in transmit SNR. Also, in Figure 11, we can see that optimal $\varepsilon^{*}$ first decrease and then slightly tends to increase with increase in transmit SNR. Finding optimal $\alpha^{*}$ and $\varepsilon^{*}$ is important to avoid an outage in the proposed system and maximizing the sum-throughput.

## 7. Conclusions and Future Works

In this paper, we presented our model on RF energy harvesting and information transmission in IoT relay systems based on time switching, power splitting and NOMA. Considering the energy constrained nature of the IoT nodes, here a power constrained IoT relay node first harvests the energy from the source node RF signal to power up themselves. The IoT relay node can harvests the energy using either time switching relaying or power splitting relaying protocol. Then in the next subsequent stage, IoT relay node transmits the source node information along with its information data using NOMA protocol. We have mathematically derived the outage probability, throughput and sum-throughput for our proposed system based on TS, PS and NOMA. Furthermore, we verified our derived analysis with the simulation results and some representative performance comparisons were presented. We showed that our analytical results for TS and PS relaying protocol exactly matched with the simulation results. We also found out the optimal time switching factor $\alpha^{*}$ and optimal power splitting factor $\varepsilon^{*}$ that maximizes the sum-throughput of the proposed system through the formulated Golden section search algorithm as shown in Algorithm 1.

For future work, we would like to investigate the ergodic capacity of the proposed system and derive the exact-forms of outage probability and sum-throughput for the proposed system. We would also like to study the performance of our proposed system by introducing interference from other nodes.

## Figures and Tables

**Table 1:** Simulation Parameters.

Parameter	Symbol	Values
Mean of ${\left|h_{IoT_{R}}\right|}^{2}\to X$	$\lambda_{h}$	1
Mean of ${\left|h_{s_{rec}}\right|}^{2}\to Y$	$\lambda_{g}$	1
Mean of ${\left|h_{IoT_{rec}}\right|}^{2}\to Z$	$\lambda_{z}$	0.5
Source Node Transmit SNR	δ	0–20 dB
Energy Harvesting Efficiency	η	1
Source and IoT Node Rate	R	1 bps/Hz
Power Factor for NOMA	$\phi_{1}$	0.2
Power Factor for NOMA	$\phi_{2}$	0.8
Noise Variance	$\sigma_{IoT_{rec}}^{2},\sigma_{s_{rec}}^{2}$	1

## Abbreviations

The following abbreviations are used in this manuscript:

**Symbol**	**Meaning**
$IoT_{R}$	IoT relay node
$P_{s}$	Power of source node transmit signal
RF	Radio Frequency
EH	Energy Harvesting
SNR	Signal-to-noise ratio
NOMA	Non-orthogonal multiple access
PS	Power splitting
DF	Decode and Forward
ϵ	Power splitting factor
$x_{s}$	Source node information data
*T*	Time period
$y_{IoT_{R}}$, ${\hat{y}}_{IoT_{R}}$	Information signal received at $IoT_{R}$
$n_{IoT_{R}}$	Additive White Gaussian Nosie at $IoT_{R}$
$\sigma_{IoT_{R}}^{2}$	Noise variance at $IoT_{R}$
$h_{IoT_{R}}$	Channel co-efficient between source node and $IoT_{R}$ node
$\lambda_{h}$	Mean variance of $h_{IoT_{R}}$
$h_{s_{rec}}$	Channel co-efficient between $IoT_{R}$ and source user
$\lambda_{g}$	Mean variance of $h_{s_{rec}}$
$h_{IoT_{rec}}$	Channel co-efficient between $IoT_{R}$ and IoT user
$\lambda_{z}$	Mean variance of $h_{IoT_{rec}}$
η	Energy conversion efficiency
$E_{h_{IoT_{R}}}$, ${\hat{E}}_{h_{IoT_{R}}}$	Energy harvested at $IoT_{R}$ node
$P_{IoT_{R}}$, ${\hat{P}}_{IoT_{R}}$	Transmit power of $IoT_{R}$ node
$Z_{I_{C1}}$, ${\hat{Z}}_{I_{C1}}$	Superimposed composite signal for NOMA protocol
$\phi_{1}$, $\phi_{2}$	Power allocation factors for NOMA protocol
$x_{IoT_{R}}$	$IoT_{R}$ node information data
$y_{s_{rec}}$, ${\hat{y}}_{s_{rec}}$	Received signal at destination source user
$y_{IoT_{rec}}$, ${\hat{y}}_{IoT_{rec}}$	Received signal at destination IoT user
$n_{s_{rec}}$, ${\hat{y}}_{IoT_{rec}}$	Additive White Gaussian Nosie at destination source user
$n_{IoT_{rec}}$	Additive White Gaussian Nosie at destination IoT user
$\gamma_{IoT_{R}}$, ${\hat{\gamma }}_{IoT_{R}}$	Received SNR at $IoT_{R}$ node
δ, $\hat{\delta }$	Transmit SNR
$\gamma_{s_{rec}}^{x_{IoT_{R}}\to x_{s}}$, ${\hat{\gamma }}_{s_{rec}}^{x_{IoT_{R}}\to x_{s}}$	SNR required at the destination source user to decode and cancel $IoT_{R}$ information data
$\gamma_{s_{rec}}$, ${\hat{\gamma }}_{s_{rec}}$	Received SNR at destination source user node
$\gamma_{IoT_{rec}}$, ${\hat{\gamma }}_{IoT_{rec}}$	Received SNR at destination $IoT_{R}$ user node
$\sigma_{s_{rec}}^{2}$	Noise variance at destination source user node
$\sigma_{IoT_{rec}}^{2}$	Noise variance at destination $IoT_{R}$ user node
ψ, $\hat{\psi }$	Outage probability
$P_{Out_{S}}$, ${\hat{P}}_{Out_{S}}$	Outage probability of source node
$P_{Out_{IoT_{R}}}$, ${\hat{P}}_{Out_{IoT_{R}}}$	Outage probability of $IoT_{R}$ node
*R*	Rate in bits per second per hertz
$Thr_{S}$, ${\hat{Thr}}_{S}$	Throughput of source node
$Thr_{IoT_{R}}$, ${\hat{Thr}}_{IoT_{R}}$	Throughput of $IoT_{R}$ node
Thr, $\hat{Thr}$	Sum-throughput of whole system
$\alpha^{*}$	Optimal time switching factor
$\varepsilon^{*}$	Optimal power splitting factor
$K_{1}\left(.\right)$	First-order modified Bessel function of the second kind
$E_{n}\left(a\right)$	Exponential integral of order n

## Appendix A. Proof of Theorem 1 in (16) and (17)

From Equation (7) we have,

$${\hat{\gamma }}_{IoT_{R}}=\hat{\delta }X\;\mathrm{where},\;{\left|h_{IoT_{R}}\right|}^{2}=X$$

Also, from Equation (9), we have,

$${\hat{\gamma }}_{s_{rec}}=\frac{\phi_{1}{\hat{P}}_{IoT_{R}}{\left|h_{s_{rec}}\right|}^{2}}{\sigma_{s_{rec}}^{2}}≜\delta XYk$$

where

$$Y={\left|h_{s_{rec}}\right|}^{2},\sigma_{s_{rec}}^{2}=1,k=\frac{2\alpha \eta P_{s}}{\left(1-\alpha \right)}$$

From Equation (11), the outage probability of the source is:

$$\begin{array}{c} {\hat{P}}_{Out_{S}}=Pr\left(min\left({\hat{\gamma }}_{IoT_{R}},{\hat{\gamma }}_{s_{rec}}\right)<\hat{\psi }\right)\\ =1-Pr(min\left({\hat{\gamma }}_{IoT_{R}},{\hat{\gamma }}_{s_{rec}}\right)\geq \hat{\psi })\\ =1-Pr(\hat{\delta }X\geq \hat{\psi },\hat{\delta }kXY\geq \hat{\psi })\\ =1-Pr(X\geq \frac{\hat{\psi }}{\hat{\delta }},Y\geq \frac{\hat{\psi }}{\delta kX}) \end{array}$$

Let

$$\begin{array}{c} x_{0}=\frac{\hat{\psi }}{\hat{\delta }},\\ =1-Pr(X\geq x_{0},Y\geq \frac{x_{0}}{kX})\\ =1-\int_{x_{0}}^{\infty }f_{X}\left(x\right)(\int_{\frac{x_{0}}{kx}}^{\infty }f_{Y}\left(y\right)dy)dx\\ =1-\int_{x_{0}}^{\infty }\lambda_{h}e^{-\lambda_{h}x}(\int_{\frac{x_{0}}{kx}}^{\infty }\lambda_{g}e^{-\lambda_{g}y}dy)dx\\ =1-\int_{x_{0}}^{\infty }\lambda_{h}e^{-\lambda_{h}x}(e^{-\lambda_{g}\frac{x_{0}}{kx}})dx\\ =1-\int_{x_{0}}^{\infty }\lambda_{h}(e^{-\lambda_{h}x-\lambda_{g}\frac{x_{0}}{kx}})dx\\ =1-\left(\underset{I_{1}}{\underset{︸}{\lambda_{h}\int_{x=0}^{\infty }(e^{-4\lambda_{g}\frac{x_{0}}{k4x}-\lambda_{h}x})dx}}-\underset{I_{2}}{\underset{︸}{\lambda_{h}\int_{x=0}^{x_{0}}(e^{-\lambda_{g}\frac{x_{0}}{kx}-\lambda_{h}x})dx}}\right) \end{array}$$

Let us first evaluate the integral $I_{1}$ by using the formula [36], Equation 3.324.1)

$$\begin{array}{c} \int_{0}^{\infty }e^{-\frac{\beta }{4x}-\gamma x}dx=\sqrt{\frac{\beta }{\gamma }}K_{1}\sqrt{\beta \gamma }\;\\ I_{1}=\lambda_{h}\sqrt{\frac{4\lambda_{g}x_{0}}{k\lambda_{h}}}K_{1}\left(\sqrt{\frac{4\lambda_{g}x_{0}\lambda_{h}}{k}}\right)\;\\ I_{1}=2\sqrt{\frac{\lambda_{h}\lambda_{g}x_{0}}{k}}K_{1}\left(2\sqrt{\frac{\lambda_{h}\lambda_{g}x_{0}}{k}}\right) \end{array}$$

Now, let us evaluate the integral $I_{2}$

$$I_{2}=\lambda_{h}\int_{x=0}^{x_{0}}(e^{-\lambda_{h}x-\frac{\lambda_{g}x_{0}}{kx}})dx$$

Expanding the term $e^{-\lambda_{h}x}$ in Taylor series

$$=\lambda_{h}\sum_{n=0}^{\infty }\frac{\left(-1\right)}{n\;!}{\left(\lambda_{h}\right)}^{n}\int_{x=0}^{x_{0}}x^{n}e^{-\frac{\lambda_{g}x_{0}}{kx}}dx$$

Substituting $y=\frac{1}{x}\to dx=-\frac{1}{y^{2}}dy$

$$=\sum_{n=0}^{\infty }\frac{\left(-1\right)}{n\;!}{\left(\lambda_{h}\right)}^{n+1}\int_{y=\frac{1}{x_{0}}}^{\infty }y^{-n-2}e^{-\frac{\lambda_{g}x_{0}y}{k}}dy$$

Now, substituting further $t=x_{0}y\to dt=x_{0}dy$

$$=\sum_{n=0}^{\infty }\frac{\left(-1\right)}{n\;!}{\left(\lambda_{h}x_{0}\right)}^{n+1}\int_{t=1}^{\infty }t^{-n-2}e^{-\frac{\lambda_{g}t}{k}}dt$$

Now, by definition of exponential integral of order n, we have,

$$\begin{array}{c} E_{n}\left(a\right)=\int_{y=1}^{\infty }y^{-n}e^{-ay}dy\\ I_{2}=\sum_{n=0}^{\infty }\frac{\left(-1\right)}{n\;!}{\left(\lambda_{h}x_{0}\right)}^{n+1}E_{n+2}\left(\frac{\lambda_{g}}{k}\right) \end{array}$$

Therefore,

$$\begin{array}{c} {\hat{P}}_{{Out}_{S}}=1-I_{1}+I_{2}\\ {\hat{P}}_{{Out}_{S}}=1-2\sqrt{\frac{\lambda_{h}\lambda_{g}x_{0}}{k}}K_{1}\left(2\sqrt{\frac{\lambda_{h}\lambda_{g}x_{0}}{k}}\right)+\sum_{n=0}^{\infty }\frac{{\left(-1\right)}^{n}}{n\;!}{\left(\lambda_{h}x_{0}\right)}^{n+1}E_{n+2}\left(\frac{\lambda_{g}}{k}\right) \end{array}$$

Putting the value of ${\hat{P}}_{{Out}_{S}}$ in Equation (13), we get,

$${\hat{Thr}}_{S}=\frac{R(1-\alpha )}{2}\left(2\sqrt{\frac{\lambda_{h}\lambda_{g}x_{0}}{k}}K_{1}\left(2\sqrt{\frac{\lambda_{h}\lambda_{g}x_{0}}{k}}\right)-\sum_{n=0}^{\infty }\frac{{\left(-1\right)}^{n}}{n\;!}{\left(\lambda_{h}x_{0}\right)}^{n+1}E_{n+2}\left(\frac{\lambda_{g}}{k}\right)\right)$$

This ends the proof of Theorem 1.

## Appendix B. Proof of Theorem 1 in (18) and (19)

From Equation (12), the outage probability of IoT relay node is:

$$\begin{array}{c} {\hat{P}}_{Out_{IoT_{R}}}=Pr\left(min\left({\hat{\gamma }}_{s_{rec}}^{x_{IoT_{R}}\to x_{s}},{\hat{\gamma }}_{IoT_{rec}}\right)<\hat{\psi }\right)\\ {\hat{P}}_{Out_{IoT_{R}}}=1-Pr(\frac{\phi_{2}lXY}{\phi_{1}lXY+1}\geq \hat{\psi },\frac{\phi_{2}lXZ}{\phi_{1}lXZ+1}\geq \hat{\psi }) \end{array}$$

where

$${\hat{P}}_{IoT_{R}}=\frac{2\alpha \eta P_{s}{\left|h_{IoT_{R}}\right|}^{2}}{\left(1-\alpha \right)}≜\frac{2\alpha \eta \hat{\delta }X}{\left(1-\alpha \right)},l=\frac{2\alpha \eta \hat{\delta }}{\left(1-\alpha \right)}$$

$$\begin{array}{c} X={\left|h_{IoT_{R}}\right|}^{2},Y={\left|h_{s_{rec}}\right|}^{2},Z={\left|h_{IoT_{rec}}\right|}^{2},\sigma_{IoT_{rec}}^{2}=1,\sigma_{s_{rec}}^{2}=1\\ =1-Pr(Y\geq \frac{\hat{\psi }}{(\phi_{2}-\phi_{1}\hat{\psi })lX},Z\geq \frac{\hat{\psi }}{(\phi_{2}-\phi_{1}\hat{\psi })lX}) \end{array}$$

Conditioning on X, we have,

$$=1-\int_{0}^{\infty }Pr(Y\geq \frac{\hat{\psi }}{(\phi_{2}-\phi_{1}\hat{\psi })lx})\times Pr(Z\geq \frac{\hat{\psi }}{(\phi_{2}-\phi_{1}\hat{\psi })lx})f_{X}\left(x\right)dx$$

put

$$\begin{array}{c} \frac{\hat{\psi }}{(\phi_{2}-\phi_{1}\hat{\psi })lx}=T\\ =1-\int_{0}^{\infty }Pr\left(Y\geq T\right)Pr\left(Z\geq T\right)f_{X}\left(x\right)dx\\ =1-\int_{0}^{\infty }(\int_{T}^{\infty }\lambda_{g}e^{-\lambda_{g}y}dy)(\int_{T}^{\infty }\lambda_{z}e^{-\lambda_{z}z}dz)\lambda_{h}e^{-\lambda_{h}x}dx\\ =1-\int_{0}^{\infty }e^{-\lambda_{g}T}e^{-\lambda_{z}T}\lambda_{h}e^{-\lambda_{h}x}dx\\ =1-\int_{0}^{\infty }e^{-\lambda_{g}T}e^{-\lambda_{z}T}\lambda_{h}e^{-\lambda_{h}x}dx \end{array}$$

substituting the value of *T* above

$$=1-\int_{0}^{\infty }e^{-\lambda_{g}\frac{\hat{\psi }}{(\phi_{2}-\phi_{1}\hat{\psi })lx}}e^{-\lambda_{z}\frac{\hat{\psi }}{(\phi_{2}-\phi_{1}\hat{\psi })lx}}\lambda_{h}e^{-\lambda_{h}x}dx$$

let

$$\begin{array}{c} d=\frac{\hat{\psi }}{(\phi_{2}-\phi_{1}\hat{\psi })l}\\ =1-\int_{0}^{\infty }e^{-\lambda_{g}\frac{d}{x}}e^{-\lambda_{z}\frac{d}{x}}\lambda_{h}e^{-\lambda_{h}x}dx\\ =1-\lambda_{h}\int_{0}^{\infty }e^{-4\left(\lambda_{g}+\lambda_{z}\right)\frac{c}{4x}-\lambda_{h}x}dx \end{array}$$

Now, using the formula

$$\begin{array}{l} \int_{0}^{\infty }e^{-\frac{\beta }{4x}-\gamma x}dx=\sqrt{\frac{\beta }{\gamma }}K_{1}\sqrt{\beta \gamma }\\ =1-\lambda_{h}\sqrt{\frac{4(\lambda_{g}+\lambda_{z})d}{\lambda_{h}}}K_{1}\left(\sqrt{4\left(\lambda_{g}+\lambda_{z}\right)d\lambda_{h}}\right)\\ {\hat{P}}_{Out_{IoT_{R}}}=1-2\sqrt{d\lambda_{h}\left(\lambda_{g}+\lambda_{z}\right)}K_{1}\left(2\sqrt{d\lambda_{h}\left(\lambda_{g}+\lambda_{z}\right)}\right) \end{array}$$

Putting the value of ${\hat{P}}_{Out_{IoT_{R}}}$ in Equation (14), we get,

$${\hat{Thr}}_{IoT_{R}}=\frac{R(1-\alpha )}{2}\left(2\sqrt{d\lambda_{h}\left(\lambda_{g}+\lambda_{z}\right)}K_{1}\left(2\sqrt{d\lambda_{h}\left(\lambda_{g}+\lambda_{z}\right)}\right)\right)$$

This ends the proof of Theorem 2.

## Appendix C. Proof of Theorem 1 in (36) and (37)

From Equation (27), we have,

$$\gamma_{IoT_{R}}=\left(1-\varepsilon \right)\delta X\;\mathrm{where}\;{\left|h_{IoT_{R}}\right|}^{2}=X$$

Also, from Equation (29), we have,

$$\gamma_{s_{rec}}=\frac{\phi_{1}P_{IoT_{R}}{\left|h_{s_{rec}}\right|}^{2}}{\sigma_{s_{rec}}^{2}}≜\delta XYa$$

where

$$Y={\left|h_{s_{rec}}\right|}^{2},\sigma_{s_{rec}}^{2}=1,a=\eta \varepsilon \phi_{1}$$

From Equation (31), the outage probability of the source is:

$$\begin{array}{c} P_{Out_{S}}=Pr\left(min\left(\gamma_{IoT_{R}},\gamma_{s_{rec}}\right)<\psi \right)\\ =1-Pr(min\left(\gamma_{IoT_{R}},\gamma_{s_{rec}}\right)\geq \psi )\\ =1-Pr((1-\varepsilon )\delta X\geq \psi ,\delta aXY\geq \psi )\\ =1-Pr(X\geq \frac{\psi }{(1-\varepsilon )\delta },Y\geq \frac{\psi }{\delta aX}) \end{array}$$

Let

$$\begin{array}{c} x_{0}=\frac{\psi }{(1-\varepsilon )\delta }\\ =1-Pr(X\geq x_{0},Y\geq \frac{\left(1-\varepsilon \right)x_{0}}{aX})\\ =1-\int_{x_{0}}^{\infty }f_{X}\left(x\right)(\int_{\frac{\left(1-\varepsilon \right)x_{0}}{ax}}^{\infty }f_{Y}\left(y\right)dy)dx\\ =1-\int_{x_{0}}^{\infty }\lambda_{h}e^{-\lambda_{h}x}(\int_{\frac{\left(1-\varepsilon \right)x_{0}}{ax}}^{\infty }\lambda_{g}e^{-\lambda_{g}y}dy)dx\\ =1-\int_{x_{0}}^{\infty }\lambda_{h}e^{-\lambda_{h}x}(e^{-\lambda_{g}\frac{\left(1-\varepsilon \right)x_{0}}{ax}})dx\\ =1-\int_{x_{0}}^{\infty }\lambda_{h}(e^{-\lambda_{h}x-\lambda_{g}\frac{\left(1-\varepsilon \right)x_{0}}{ax}})dx\\ =1-\left(\underset{I_{1}}{\underset{︸}{\lambda_{h}\int_{x=0}^{\infty }(e^{-4\lambda_{g}\frac{\left(1-\varepsilon \right)x_{0}}{a4x}-\lambda_{h}x})dx}}-\underset{I_{2}}{\underset{︸}{\lambda_{h}\int_{x=0}^{x_{0}}(e^{-\lambda_{g}\frac{\left(1-\varepsilon \right)x_{0}}{ax}-\lambda_{h}x})dx}}\right) \end{array}$$

Let us first evaluate the integral $I_{1}$ by using the formula [36], Equation 3.324.1)

$$\begin{array}{c} \int_{0}^{\infty }e^{-\frac{\beta }{4x}-\gamma x}dx=\sqrt{\frac{\beta }{\gamma }}K_{1}\left(\sqrt{\beta \gamma }\right)\\ I_{1}=\lambda_{h}\sqrt{\frac{4\lambda_{g}\left(1-\varepsilon \right)x_{0}}{a\lambda_{h}}}K_{1}\left(\sqrt{\frac{4\lambda_{g}\left(1-\varepsilon \right)x_{0}\lambda_{h}}{a}}\right)\\ I_{1}=2\sqrt{\frac{\lambda_{h}\lambda_{g}\left(1-\varepsilon \right)x_{0}}{a}}K_{1}\left(2\sqrt{\frac{\lambda_{h}\lambda_{g}\left(1-\varepsilon \right)x_{0}}{a}}\right) \end{array}$$

Now, let us evaluate the integral $I_{2}$

$$I_{2}=\lambda_{h}\int_{x=0}^{x_{0}}(e^{-\lambda_{h}x-\frac{\left(1-\varepsilon \right)\lambda_{g}x_{0}}{ax}})dx$$

Expanding the term $e^{-\lambda_{h}x}$ in Taylor series

$$=\lambda_{h}\sum_{n=0}^{\infty }\frac{\left(-1\right)}{n\;!}{\left(\lambda_{h}\right)}^{n}\int_{x=0}^{x_{0}}x^{n}e^{-\frac{\left(1-\varepsilon \right)\lambda_{g}x_{0}}{ax}}dx$$

Substituting $y=\frac{1}{x}$, and further $t=x_{0}y$, we get,

$$=\sum_{n=0}^{\infty }\frac{\left(-1\right)}{n\;!}{\left(\lambda_{h}x_{0}\right)}^{n+1}\int_{t=1}^{\infty }t^{-n-2}e^{-\frac{\left(1-\varepsilon \right)\lambda_{g}t}{a}}dt$$

Now, by definition of exponential integral of order *n*, we have

$$\begin{array}{c} E_{n}\left(a\right)=\int_{y=1}^{\infty }y^{-n}e^{-ay}dy\\ I_{2}=\sum_{n=0}^{\infty }\frac{\left(-1\right)}{n\;!}{\left(\lambda_{h}x_{0}\right)}^{n+1}E_{n+2}\left(\frac{\left(1-\varepsilon \right)\lambda_{g}}{a}\right) \end{array}$$

Therefore,

$$P_{{Out}_{S}}=1-2\sqrt{\frac{\lambda_{h}\lambda_{g}\left(1-\varepsilon \right)x_{0}}{a}}K_{1}\left(2\sqrt{\frac{\lambda_{h}\lambda_{g}\left(1-\varepsilon \right)x_{0}}{a}}\right)+\sum_{n=0}^{\infty }\frac{{\left(-1\right)}^{n}}{n\;!}{\left(\lambda_{h}x_{0}\right)}^{n+1}E_{n+2}\left(\frac{\left(1-\varepsilon \right)\lambda_{g}}{a}\right)$$

Putting the value of $P_{{Out}_{S}}$ in Equation (33), we get,

$$Thr_{S}=\frac{R}{2}\left(2\sqrt{\frac{\lambda_{h}\lambda_{g}\left(1-\varepsilon \right)x_{0}}{a}}K_{1}\left(2\sqrt{\frac{\lambda_{h}\lambda_{g}\left(1-\varepsilon \right)x_{0}}{a}}\right)-\sum_{n=0}^{\infty }\frac{{\left(-1\right)}^{n}}{n\;!}{\left(\lambda_{h}x_{0}\right)}^{n+1}E_{n+2}\left(\frac{\left(1-\varepsilon \right)\lambda_{g}}{a}\right)\right)$$

This ends the proof of Theorem 3.

## Appendix D. Proof of Theorem 1 in (38) and (39)

From Equation (32), the outage probability of IoT relay node is:

$$\begin{array}{c} P_{Out_{IoT_{R}}}=Pr\left(min\left(\gamma_{s_{rec}}^{x_{IoT_{R}}\to x_{s}},\gamma_{IoT_{rec}}\right)<\psi \right)\\ P_{Out_{IoT_{R}}}=1-Pr(\frac{\phi_{2}bXY}{\phi_{1}bXY+1}\geq \psi ,\frac{\phi_{2}bXZ}{\phi_{1}bXZ+1}\geq \psi ) \end{array}$$

where

$$\begin{array}{c} P_{IoT_{R}}=\eta \varepsilon P_{s}{\left|h_{IoT_{R}}\right|}^{2}≜\eta \varepsilon \delta X,b=\eta \delta \varepsilon \\ X={\left|h_{IoT_{R}}\right|}^{2},Y={\left|h_{s_{rec}}\right|}^{2},Z={\left|h_{IoT_{rec}}\right|}^{2},\sigma_{IoT_{rec}}^{2}=1,\sigma_{s_{rec}}^{2}=1\\ =1-Pr(Y\geq \frac{\psi }{(\phi_{2}-\phi_{1}\psi )bX},Z\geq \frac{\psi }{(\phi_{2}-\phi_{1}\psi )bX}) \end{array}$$

Conditioning on *X*, we have,

$$=1-\int_{0}^{\infty }Pr(Y\geq \frac{\psi }{(\phi_{2}-\phi_{1}\psi )bx})\times Pr(Z\geq \frac{\psi }{(\phi_{2}-\phi_{1}\psi )bx})f_{X}\left(x\right)dx$$

putting,

$$\begin{array}{c} \frac{\psi }{(\phi_{2}-\phi_{1}\psi )bx}=U\\ =1-\int_{0}^{\infty }Pr\left(Y\geq U\right)Pr\left(Z\geq U\right)f_{X}\left(x\right)dx\\ =1-\int_{0}^{\infty }\left(\int_{U}^{\infty }\lambda_{g}e^{-\lambda_{g}y}dy\right)\left(\int_{U}^{\infty }\lambda_{z}e^{-\lambda_{z}z}dz\right)\lambda_{h}e^{-\lambda_{h}x}dx\\ =1-\int_{0}^{\infty }e^{-\lambda_{g}U}e^{-\lambda_{z}U}\lambda_{h}e^{-\lambda_{h}x}dx \end{array}$$

substituting the value of *U* above

$$=1-\int_{0}^{\infty }e^{-\lambda_{g}\frac{\psi }{(\phi_{2}-\phi_{1}\psi )bx}}e^{-\lambda_{z}\frac{\psi }{(\phi_{2}-\phi_{1}\psi )bx}}\lambda_{h}e^{-\lambda_{h}x}dx$$

let

$$\begin{array}{c} c=\frac{\psi }{(\phi_{2}-\phi_{1}\psi )b}\\ =1-\int_{0}^{\infty }e^{-\lambda_{g}\frac{c}{x}}e^{-\lambda_{z}\frac{c}{x}}\lambda_{h}e^{-\lambda_{h}x}dx\\ =1-\lambda_{h}\int_{0}^{\infty }e^{-4\left(\lambda_{g}+\lambda_{z}\right)\frac{c}{4x}-\lambda_{h}x}dx \end{array}$$

Now, using the formula,

$$\begin{array}{c} \int_{0}^{\infty }e^{-\frac{\beta }{4x}-\gamma x}dx=\sqrt{\frac{\beta }{\gamma }}K_{1}\left(\sqrt{\beta \gamma }\right)\\ =1-\lambda_{h}\sqrt{\frac{4(\lambda_{g}+\lambda_{z})c}{\lambda_{h}}}K_{1}\left(\sqrt{4\left(\lambda_{g}+\lambda_{z}\right)c\lambda_{h}}\right)\\ P_{Out_{IoT_{R}}}=1-2\sqrt{c\lambda_{h}\left(\lambda_{g}+\lambda_{z}\right)}K_{1}\left(2\sqrt{c\lambda_{h}\left(\lambda_{g}+\lambda_{z}\right)}\right) \end{array}$$

Putting the value of $P_{Out_{IoT_{R}}}$ in Equation (34), we get,

$$Thr_{IoT_{R}}=\frac{R}{2}\left(2\sqrt{c\lambda_{h}\left(\lambda_{g}+\lambda_{z}\right)}K_{1}\left(2\sqrt{c\lambda_{h}\left(\lambda_{g}+\lambda_{z}\right)}\right)\right)$$

This ends the proof of Theorem 4.
